# Fat, adipokines, bone structure and bone regulatory factors associations in obesity

**DOI:** 10.1530/EJE-22-0530

**Published:** 2022-09-29

**Authors:** T Vilaca, A Evans, F Gossiel, M Paggiosi, R Eastell, J S Walsh

**Affiliations:** 1 Mellanby Centre for Bone Research, Department of Oncology and Metabolism, University of Sheffield, Sheffield, UK

## Abstract

**Context:**

Obese (OB) adults (BMI ≥ 30) have a higher bone mineral density (BMD) and more favourable bone microarchitecture than normal-weight (NW) adults (BMI 18.5–24.9).

**Objective:**

The objective of this study was to identify which fat compartments have the strongest association with bone density and bone turnover and whether biochemical factors (adipokines, hormones and bone regulators) are likely to be important mediators of the effect of obesity on bone.

**Design:**

This was a cross-sectional, observational, matched case-control study.

**Setting:**

Participants were recruited from the local community.

**Participants:**

Two hundred healthy men and women aged 25–40 or 55–75 were recruited in individually matched OB and NW pairs. Body composition, BMD and bone microarchitecture were determined by dual-energy X-ray absorptiometry (DXA), computed tomography (CT) and high-resolution peripheral CT (HR-pQCT). Bone turnover and potential regulators such as C-terminal cross-linking telopeptide (CTX), type 1 procollagen N-terminal peptide (PINP), sclerostin, periostin, parathyroid hormone (PTH), 25-hydroxyvitamin D (25OHD), insulin-like growth factor 1 (IGF1), adiponectin, leptin and insulin were assessed.

**Main outcome:**

Planned exploratory analysis of the relationships between fat compartments, areal and volumetric BMD, bone microarchitecture, bone turnover markers and bone regulators.

**Results:**

Compared with NW, OB had lower CTX, PINP, adiponectin, IGF1, and 25OHD and higher leptin, PTH and insulin (all *P* < 0.05). CTX and subcutaneous adipose tissue (SAT) were the bone marker and fat compartment most consistently associated with areal and volumetric BMD. In regression models, SAT was negatively associated with CTX (*P* < 0.001). When leptin was added to the model, SAT was no longer associated with CTX, but leptin (*P* < 0.05) was negatively associated with CTX.

**Conclusions:**

SAT is associated with lower bone resorption and properties favourable for bone strength in obesity. Leptin may be an important mediator of the effects of SAT on the skeleton.

## Introduction

Obesity is a global epidemic. Despite generally detrimental health effects, higher body weight and body mass index (BMI) are associated with higher bone mineral density (BMD) and lower risk of hip and vertebral fractures (1, 2), but higher risk of foot, ankle and humerus fractures (3, 4). We have previously shown higher bone density, favourable bone microarchitecture and greater bone strength in obese (OB) adults compared to normal-weight (NW) adults (5). Cortical thickness and density were greater in OB, but cortical perimeter did not differ. Both endocrine and mechanical stimuli might contribute to these findings, but because mechanical loading is known to increase bone size, we hypothesised that endocrine effects are likely to be important mediators of the bone phenotype in obesity.

There is evidence to support complex crosstalk between bone, adipocytes, osteokines, cytokines, hormones and other biochemical factors in obesity (6). Several regulatory factors might be involved, such as leptin, adiponectin, periostin and sclerostin. Leptin is produced by adipocytes in proportion to fat mass, and experimental models suggest that it has complex effects on bone (7). It acts through hypothalamic relays to increase sympathetic tone and therefore increase bone resorption. In contrast, in peripheral circulation, it seems to increase bone formation through direct effects on osteoblasts. Therefore, leptin could have negative, positive or neutral effects on bone in obesity, depending on the balance of these actions (7). Adiponectin is also produced by adipocytes, in inverse proportion to fat mass. In most clinical observations, adiponectin is inversely associated with bone mass, although its mechanism of action on bone is not yet clearly characterised (8). Periostin is an extracellular matrix protein broadly expressed. In bone, the expression levels are maximal in the periosteum. It increases bone formation through osteoblast differentiation, cell adhesion, Wnt signalling and collagen cross-linking (9, 10, 11). Periostin expression increases with skeletal loading and intermittent parathyroid hormone (PTH) treatment (12). Therefore, it is a potential mediator of loading effects of obesity on bone. In a cross-sectional study in young women, periostin was lower in OB compared to NW controls and women with anorexia nervosa (13); however, there are no published data on how periostin relates to bone strength in obesity. Finally, sclerostin is a secreted protein that inhibits Wnt-inducible osteogenesis (14). It is produced by osteocytes and plays a central role in the anabolic response of bone to mechanical loading (14). The contribution of each of these factors to the favourable bone profile in obesity is unknown.

Furthermore, each fat compartment has a specific metabolic profile, resulting in different effects on bone. For example, there is evidence that subcutaneous fat has greater expression of leptin (which would be favourable to bone), while visceral fat is pro-inflammatory (which would be detrimental to bone) (15). The net effect of obesity on the bone will depend on the balance of the effects of these different compartments.

In summary, associations between adipocytes, osteokines, cytokines, hormones and bone turnover, bone density and microstructure might explain how adiposity exerts positive effects on bone density and microstructure. The aims of this study are to determine: (i) which fat compartments have the strongest association with bone density and bone turnover and (ii) the biochemical factors which might mediate this relationship.

## Methods

### Study design and participants

As previously reported (5), we conducted a cross-sectional study of 200 OB and NW community-dwelling men and women from South Yorkshire, UK, aged 25–40 years (*n* = 80) or 55–75 years (*n* = 120). Participants were recruited through general practitioners, university and hospital staff and students, and poster advertisements. OB individuals (BMI ≥ 30 kg/m^2^) were individually matched to NW individuals (BMI 18.5–24.9 kg/m^2^) by sex, age (±3 years), height (±5 cm), smoking status (current smoker or non-smoker) and postcode.

All women aged 25–40 years were pre-menopausal, and those aged 55–75 years were at least 5 years post-menopausal. Participants were excluded if they had pre-diagnosed conditions (including diabetes) or were taking medications known to affect bone metabolism (women using hormonal contraceptives and hormone replacement therapy were excluded), had fractured or undergone orthopaedic surgery within the last 12 months, were highly physically active (≥7 h per week), consumed above 21 units of alcohol per week or were actively trying to lose weight.

All participants provided written informed consent and the study was conducted according to the Declaration of Helsinki. Ethical approval was obtained from Sheffield Research Ethics Committee.

### Body weight and fat measures

Height (cm) and weight (kg) were measured using a wall-mounted stadiometer (Seca 242, Seca, Birmingham, UK) and an electronic balance scale (Seca). BMI was calculated as weight kg/(height m)^2^.

Whole-body fat mass (FM), trunk FM, android FM, gynoid FM and appendicular FM were determined by whole-body dual-energy X-ray absorptiometry (DXA; Discovery A, Hologic Inc, Bedford, MA, USA). Total abdominal, abdominal subcutaneous adipose tissue (SAT) and abdominal visceral adipose tissue (VAT) volumes were determined by five-slice CT, taken at the mid-level of the L3 vertebra (LightSpeed VCT-XT, General Electric Healthcare). Volumes were determined using the Volume Viewer imaging software (General Electric Healthcare). The middle axial slice in the image sequence was selected. To identify adipose tissue, a threshold of −30 to −130 HU was applied. The total volume of adipose tissue was calculated using the histogram function. A manual trace function was applied inside the SAT inner border and the ‘cut outside’ function was applied to remove SAT from the image. The histogram function was re-applied to quantify the remaining VAT, and SAT was determined as total minus VAT.

### Bone structure

Bone density (g/cm^2^) at the whole body, lumbar spine (LS) and total hip were measured by DXA (Hologic Discovery A; Hologic).

Distal radius and distal tibia were scanned by XtremeCT device (Scanco Medical AG, Zurich, Switzerland) using standard protocols. Images were analysed with the standard software and extended cortical measures software provided by Scanco Medical AG (version 6).

### Biochemistry

Blood samples were collected between 08:00 and 10:00 h following an overnight fast. Samples were allowed to clot at room temperature for 30 min, centrifuged at 1008 ***g*** for 10 min and stored at −80°C until analysis. Calcium, albumin, PTH, glucose, insulin, 25-hydroxyvitamin D (25OHD), collagen type 1 C-telopeptide (CTX) and type I procollagen N-terminal peptide (PINP) were measured with autoanalysers (Cobas c701, c702, e411 and e602, Roche Diagnostics). The inter-assay precision was <6.0% for all tests. Insulin-like growth factor (IGF1) was measured by automated CLIA (IDS-iSYS, Immunodiagnostic Systems, Boldon, UK; inter-assay CV all <6.0%). Leptin and adiponectin were measured with manual ELISA (Quantikine, R&D Systems; inter-assay CV 3.8% for leptin and 2.8% for adiponectin). Sclerostin and periostin were measured by manual ELISA (Biomedica, Vienna, Austria) with inter-assay CV 9.1% and 6.0%, respectively.

### Statistical analysis

The sample size for the study was originally calculated to detect a 7.5% difference in hip DXA BMD between OB and NW, with 80% power at *P* < 0.05 using a paired-sample t-test. All variables were assessed for normality and log transformed where necessary. Paired sample t-tests were used to determine significant differences between NW and OB groups. Wilcoxon signed-rank test was used where the transformed values remained non-normal. Univariate general linear models were used to identify whether age group, gender and BMI influenced FM or biochemical outcomes and to identify interactions between age or gender and the effect of BMI on FM or biochemical measurements. Pearson correlation coefficient was used to determine associations between FM and biochemical variables, FM and skeletal outcomes and biochemistry and skeletal outcomes. Spearman’s rank correlation coefficient was used where the sample distribution remained non-normal. Multiple linear regression, adjusting for age and gender, was used to determine whether FM or lean mass (LM) predicted bone density and microstructure. Whole body lean mass (WBLM) and each FM variable separately were entered as independent variables to avoid collinearity. Multiple linear regression models were constructed to determine the influence of different fat compartments on areal BMD (aBMD) and volumetric BMD (vBMD). aBMD and vBMD were entered as the dependent variables, and age, gender and pairs of contrasting fat compartments (SAT and VAT, trunk FM and appendicular FM, android FM and gynoid FM) were entered as independent variables. To determine the influence of different fat compartments on cortical and trabecular outcomes, multiple linear regression with microstructural outcome as the dependent variable and age, gender and pairs of contrasting fat compartments (SAT and VAT, trunk FM and appendicular FM, and android FM and gynoid FM) as independent variables was performed.

First, the fat compartment with the strongest and most consistent relationship with bone turnover marker was identified. We then investigated which candidate hormones and regulators of bone turnover differed between OB and NW, and which of these were most strongly associated with bone turnover. Then, adipokines and bone regulators were successively added to the regression model including fat mass, age and gender. Analysis was performed using IBM SPSS Statistics for Windows (Version 21.0, IBM Corp). Significance was accepted when *P* < 0.05.

## Results

**Figure 1:**
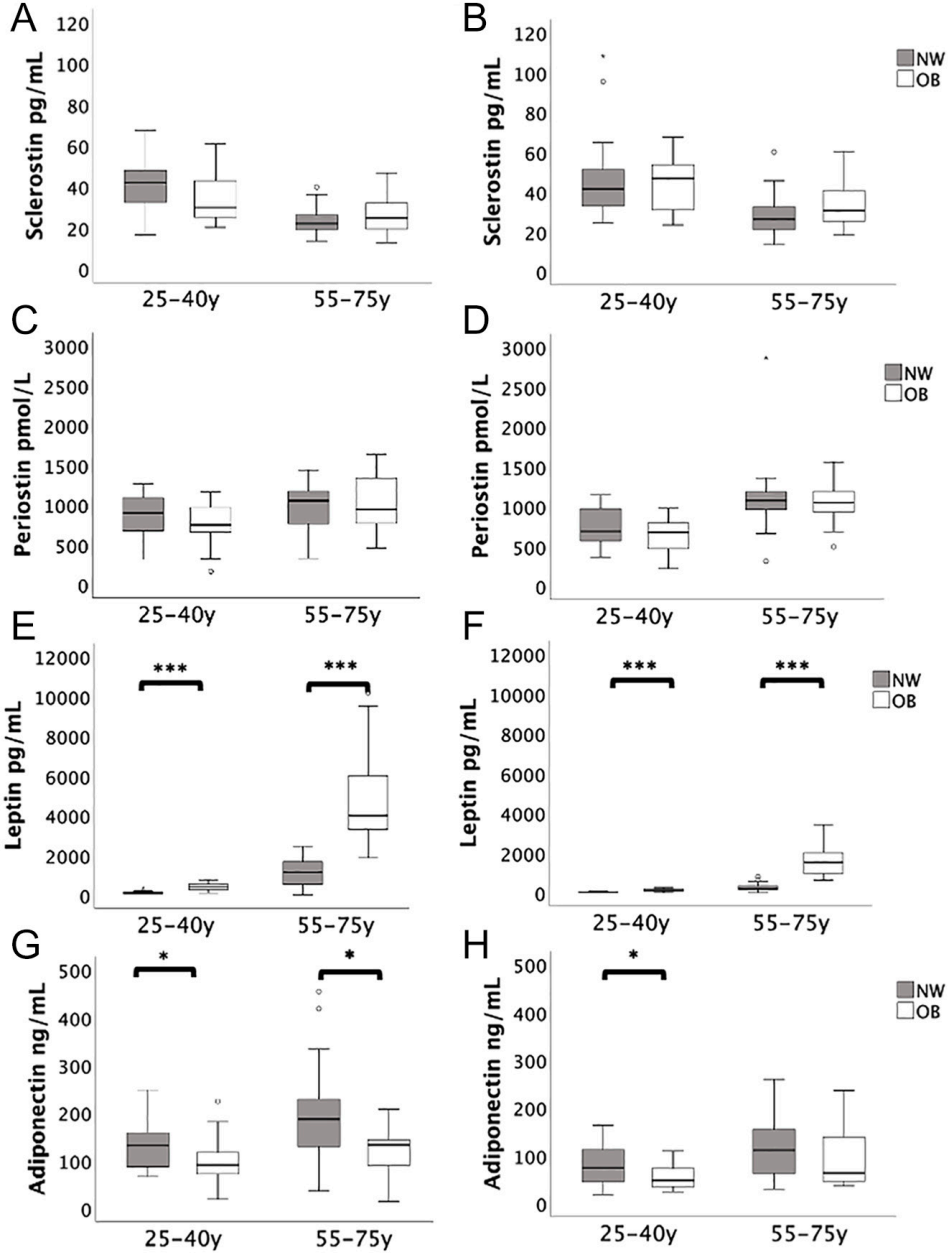
Sclerostin, periostin, leptin and adiponectin by age and gender (women on the left, men on the right) *P < 0.05; **P < 0.01; ***P < 0.001.

**Figure 2:**
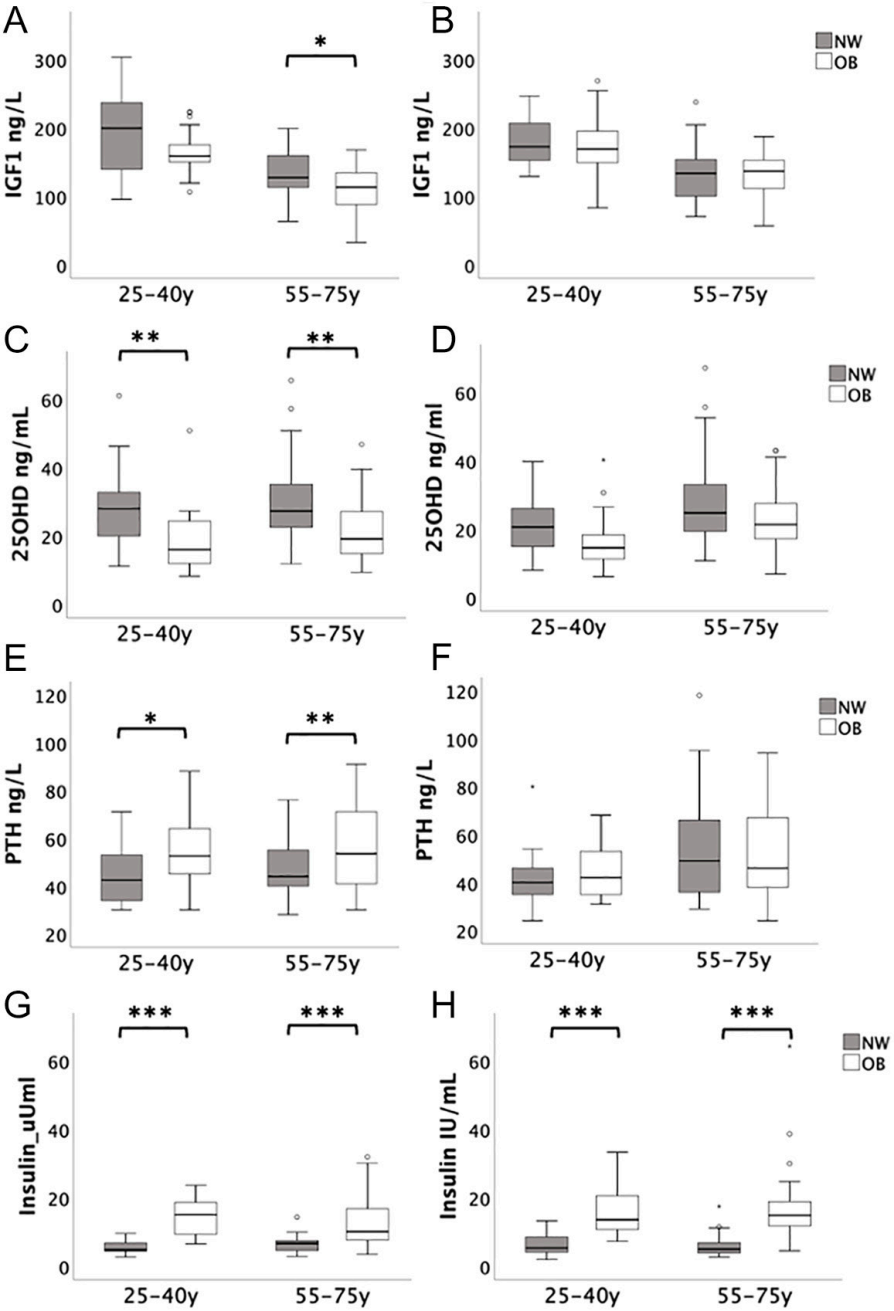
IGF1, 25OHD, PTH and insulin by age and gender (women on the left, men on the right) *P < 0.05; **P < 0.01; ***P < 0.001.

Participant characteristics are given in Table 1. There was no difference between OB and NW for sclerostin or periostin (showed by each age group in Fig. 1A, B, C and D). OB people had higher leptin (*P* < 0.001) (Fig. 1E and F) and lower adiponectin (*P* < 0.001) (Fig. 1G and H) compared to NW people. OB people also had lower IGF1 (*P* < 0.05) (Fig. 2A and B), and 25OHD (*P* < 0.001) (Fig. 2C and D) and higher PTH (*P* < 0.05) (Fig. 2E and F), insulin (Fig. 2G and H), glucose, homeostatic model assessment for insulin resistance (HOMA-IR) (all *P* < 0.001) than NW people. There was no difference between OB and NW people for calcium. The analyses excluding participants with glucose > 7 mmol/L (*n* = 7 all in the OB group) or vitamin D < 12 nmol/L (20 in OB and 5 in NW) led to similar results.

**Table 1:** Participants’ characteristics. Values are given as mean (sd.).

	Women				Men			
	25–40 years (*n* = 44)		55–75 years (*n* = 60)		25–40 years (*n* = 36)		55–75 years (*n* = 60)	
	Normal	Obese	Normal	Obese	Normal	Obese	Normal	Obese
BMI, kg/cm^2^	22.4 (1.5)***	35.4 (4.2)***	22.9 (1.5)***	35.9 (5.0)***	23.0 (1.3)***	32.9 (2.6)***	23.4 (1.2)***	34.6 (4.0)***
Height, cm	167.1 (6.8)	164.5 (7.3)	161.1 (4.3)	160.6 (4.0)	178.4 (7.0)	180.4 (6.7)	175.5 (6.4)	176.1 (7.5)
Whole body fat mass, kg	19.4 (2.7)***	42.8 (8.5)***	20.3 (3.6)***	41.9 (9.5)***	14.9 (2.7)***	35.0 (5.6)***	16.6 (2.9)***	36.6 (7.6)***
Whole body lean mass, kg	39.1 (4.1)***	48.8 (6.8)***	35.2 (3.7)***	46.3 (4.6)***	53.9 (6.7)***	66.7 (7.9)***	50.7 (4.8)***	65.0 (6.7)***
SAT, g/cm^3^	35.9 (11.1)***	110.6 (27.7)***	42.6 (10.5)***	98.3 (29.6)***	26.3 (8.4)***	79.2 (21.0)***	34.2 (9.2)***	79.3 (24.1)***
VAT, g/cm^3^	12.1 (5.5)***	40.8 (10.0)***	23.0 (9.2)***	48.6 (16.8)***	21.5 (7.8)***	53.5 (16.1)***	34.8 (14.0)***	80.7 (15.8)***
VAT/SAT ratio	0.34 (0.1)	0.38 (0.1)	0.54 (0.2)	0.53 (0.2)	0.85 (0.3)	0.71 (0.2)	1.06 (0.5)	1.12 (0.4)
Whole body aBMD, g/cm^2^	1.112 (0.09)	1.116 (0.06)	1.006 (0.10)**	1.129 (0.13)**	1.166 (0.08)	1.211 (0.10)	1.100 (0.10)**	1.195 (0.13)**
Lumbar spine aBMD, g/cm^2^	1.049 (0.14)	1.097 (0.10)	0.888 (0.11)***	1.090 (0.12)***	1.007 (0.11)*	1.086 (0.12)*	0.958 (0.13)***	1.123 (0.16)***
Total hip aBMD, g/cm^2^	0.940 (0.09)***	1.060 (0.08)***	0.810 (0.07)***	1.010 (0.12)***	0.990 (0.13)**	1.120 (0.12)**	0.930 (0.14)***	1.100 (0.15)***
Distal tibia vBMD, mgHA/cm^3^	308.9 (36.3)*	336.3 (47.6)*	255.0 (42.5)***	316.8 (49.8)***	332.5 (36.6)***	378.6 (31.3)***	283.9 (49.9)*	328.0 (50.9)*
Distal tibia cortical vBMD, mgHA/cm^3^	998 (33)	1004 (28)	837 (80)***	922 (62)***	974 (50)	970 (35)	887 (82)	914 (57)
Distal tibia cortical thickness, mm^a^	1.16 (0.2)*	1.30 (0.22)*	0.81 (0.23)***	1.16 (0.2)***	1.37 (0.25)*	1.54 (0.22)*	1.08 (0.25)**	1.30 (0.31)
Distal tibia trabecular vBMD, mgHA/cm^3^	172 (27)	187 (32)	167 (31)***	197 (37)***	198 (29)***	238 (22) ***	181 (33)**	205 (36)**
Distal tibia trabecular number, 1/mm	1.87 (0.30)*	2.07 (0.28)*	1.79 (0.30)***	2.09 (0.36)***	1.88 (0.29)***	2.30 (0.26)***	1.81 (0.21)***	2.24 (0.34)***
Distal radius vBMD, mgHA/cm^3^	293.6 (56.4)	312.1 (45.7)	259.7 (63.3)***	326.2 (47.9)***	325.8 (37.0)	336.9 (40.0)	273.9 (55.8)**	324.1 (50.7)**
Distal radius cortical vBMD, mgHA/cm^3^	1013 (34)	1014 (31)	940 (62)*	975 (50)*	981 (37)	960 (38)	951 (54)	932 (104)
Distal radius cortical thickness, mm^a^	0.69 (0.15)	0.75 (0.12)	0.53 (0.21)***	0.73 (0.15)***	0.75 (0.18)	0.79 (0.17)	0.65 (0.21)**	0.78 (0.17)**
Distal radius trabecular vBMD, mgHA/cm^3^	150 (32)	164 (29)	142 (30)***	179 (37)***	194 (26)	210 (22)	163 (29)***	201 (36)***
Distal radius trabecular number, 1/mm	1.94 (0.34)*	2.11 (0.17)*	1.81 (0.32)***	2.21 (0.34)***	2.06 (0.28)*	2.28 (0.20)*	2.02 (0.24)***	2.30 (0.24)***
CTX, ng/mL	0.329 (0.10)	0.299 (0.10)	0.441 (0.18)**	0.328 (0.10)**	0.476 (0.13)*	0.385 (0.14)*	0.381 (0.15)**	0.270 (0.14)**
PINP, ng/mL	43.5 (12.5)	39.7 (13.4)	51.7 (19.7)*	41.7 (12.2)*	66.6 (36.5)	52.7 (21.2)	42.5 (18.7)	35.1 (14.9)

^a^Derived measurement method (Whittier *et al.* (29)). *P < 0.05; **P < 0.01; ***P < 0.001 (comparison of OB vs NW in each age and gender group).

aBMD, areal bone mineral density; CTX, C-terminal cross-linking telopeptide; PINP, type 1 procollagen N-terminal peptide; SAT, subcutaneous abdominal tissue; VAT, visceral abdominal tissue; vBMD, volumetric bone mineral density.

Leptin was higher in older adults than younger adults and higher in women than in men, and these differences persisted after correction for whole-body FM (both *P* < 0.001) (Fig. 1E and F). There was a significant age interaction with BMI effect on leptin, such that the effect of BMI on leptin was greater in older adults (*P* < 0.05).

The relationships between fat compartments, areal and volumetric BMD are given in Table 2. All the statistically significant associations between fat and bone structural measures were positive. SAT was the compartment most consistently associated with bone measures. It was significantly associated with DXA aBMD of spine and hip, high-resolution peripheral CT (HR-pQCT) vBMD of radius and tibia and it was the only fat compartment significantly associated with HR-pQCT microarchitecture of the radius and tibia (Table 3). Therefore, we took SAT forward into the exploratory mechanistic models.

**Table 2:** Fat compartment associations with areal and volumetric bone density by multiple linear regression, adjusted for age and gender.

	SAT	VAT	Model adjusted *R*^2^	Android FM	Gynoid FM	Model adjusted *R*^2^	AFM	TFM	Adjusted *R*^2^
	Beta	Beta		Beta	Beta		Beta	Beta	
aBMD									
WB	0.356***	-0.26	0.341***	−0.68	0.474***	0.361***	0.553***	−0.14	0.37***
TH	0.418***	0.096	0.336***	0.23	0.341*	0.386***	0.300*	0.263	0.384***
LS	0.328**	0.096	0.155***	0.065	0.433**	0.225***	0.279	0.188	0.202***
vBMD									
Radius	0.288*	0.079	0.139***	0.299*	0.059	0.147***	0.015	0.326*	0.139***
Tibia	0.342**	0.058	0.306***	0.407**	−0.015	0.320***	−0.083	0.452**	0.310***

*P < 0.05; **P < 0.01; ***P < 0.001.

aBMD, areal bone mineral density; AFM, appendicular fat mass; FM, fat mass; LS, lumbar spine; SAT, subcutaneous abdominal tissue; TFM, trunk fat mass; TH, total hip; VAT, visceral abdominal tissue; vBMD, volumetric bone mineral density.

**Table 3:** SAT associations with bone microarchitecture at the radius and tibia by multiple linear regression.

Variable	Radius			Tibia		
	Beta	*P* value	Adjusted *R*^2^	Beta	*P* value	Adjusted *R*^2^
Ct.vBMD	0.245	0.035	0.119***	NS	NS	NS
Ct.Ar	0.350	0.001	0.318***	0.302	0.001	0.471***
Ct.Th	0.303	0.009	0.125***	0.290	0.004	0.338***
Tb.vBMD	0.253	0.014	0.307***	0.331	0.002	0.225***
Tb.N	0.428	<0.001	0.241***	0.604	<0.001	0.250***

*P < 0.05; **P < 0.01; ***P < 0.001.

CTX was consistently decreased in OB compared to NW (Table 1). Therefore, we hypothesised that decreased resorption is the main reason for higher BMD in obesity. Leptin was negatively associated with CTX (regression model beta: 0.288, *P* < 0.001; adjusted *r* ^2:^ 0.078, *P* = 0.01). However, when leptin was added to the model, SAT became non-significant (*P* = 0.64), suggesting that the effect of SAT on CTX is mediated through leptin (beta: −0.409, *P* < 0.05; adjusted *r* ^2:^ 0.101, *P* < 0.001). These findings suggest that SAT effect on CTX is associated with leptin.

## Discussion

We investigated the differences in adipokines and bone regulators between OB and NW people. As expected, leptin, insulin, glucose, HOMA-IR and PTH were higher in OB people, and adiponectin, IGF1 and 25OHD were lower. No difference was observed in calcium, sclerostin and periostin. We explored the relationship between fat compartments and bone measures and determined that SAT was the compartment most consistently associated with bone density and structure. SAT was associated with vBMD and microarchitecture at the radius and tibia, and aBMD at the LS and proximal femur. CTX, a bone resorption marker, was lower in OB people suggesting that decreased resorption is an important mechanism for higher BMD in obesity. Regression models suggested that leptin is a key factor in the association of SAT with CTX. Our results suggest that endocrine mechanisms play a role in the bone strength of OB adults. We propose that peripheral leptin inhibits bone resorption in obesity.

In contrast, our findings did not show associations with bone regulators associated with mechanical loading. We have shown that cortical thickness and density are greater in OB adults with no difference in cortical perimeter (5), and mechanical loading affects cortical perimeter (16). Also, periostin and sclerostin which are bone regulators strongly associated with loading did not differ between NW and OB and were not associated with bone measures in obesity. Finally, the similar bone measures at the tibia and the non-weight bearing radius support the hypothesis that this relationship is driven by hormonal rather than biomechanical effects of weight.

SAT was consistently associated with bone density (both volumetric and areal) and microarchitecture, suggesting a role for SAT in the favourable bone properties observed in obesity. SAT is the fat beneath the skin of the abdomen but above the abdominal muscle wall, whereas VAT accumulates beneath the abdominal muscle wall, around the organs (17). SAT expresses more leptin, while VAT expresses more pro-inflammatory cytokines (18, 19).

Our results suggest that the anti-resorptive action of leptin in the peripheral skeleton is the dominant effect of this adipokine at the skeleton in obesity. Leptin was higher in OB than NW and was a negative predictor of CTX. Leptin is produced by adipocytes and has an important role in regulating appetite and energy homeostasis via actions on the hypothalamus (20). Leptin is a marker of fat reserves and circulating levels are high in obesity (7). *In vitro* studies have shown that leptin enhances osteoblastic differentiation and regulates osteoclastogenesis, inhibiting RANKL (21). Conversely, animal studies have shown that leptin action in the CNS has indirect catabolic effects on bone, mediated through beta-adrenoreceptors (21). Therefore, peripheral leptin has anabolic effects on bone while central leptin has catabolic effects. Preclinical studies also suggest that the direct effect of leptin on bone is likely to be dominant (7).

Robust evidence supports a positive association between BMI and aBMD (5, 22), but less evidence is available regarding BMI with vBMD and microarchitecture. Most of the studies explored overall FM but not fat compartments and few investigated the effect of SAT on bone features (22, 23, 24). In the OFELY cohort, favourable microarchitecture was reported in OB women (25). Data from the Framingham study suggested that bone strength was greater in those with larger amounts of VAT despite some deleterious effects of VAT on cortical bone. However, SAT effects on microarchitecture were not assessed (24). Conversely, Gilsanz *et al*. used CT to evaluate healthy young women and reported that visceral and subcutaneous fat have opposite effects on the appendicular skeleton; whereas subcutaneous fat was beneficial to bone structure and strength, visceral fat had negative associations with bone features (26). In addition, a recent study including 4900 healthy individuals aged 30–50 years old reported that VAT in both men and women was associated with lower aBMD (27). Ng *et al*. assessed central and peripheral vBMD in a cohort of men and women aged 20–97 years (28). In agreement with our results, they found positive correlations of SAT with vBMD and microarchitecture. These results agree with our findings of SAT being the main fat compartment associated with favourable bone features in obesity.

A number of studies reported that the associations between bone features and fat compartments became attenuated or non-significant after adjusting for BMI (24, 25, 28). Some researchers suggest that the effect of attenuation by adjustment for weight implies that the effect of FM is due to mechanical loading on the skeleton (24). However, adipokines are also quantitatively associated with weight.

This study has limitations. This is a cross-sectional observational study; therefore, we can report associations, but we cannot infer causation. In addition, the study was originally powered to detect differences in BMD and BTM between OB and NW and the present study is a planned exploratory analysis. The strengths of the study are that data came from both men and women, young and old, NW, and OB and individually matched pairs.

In summary, we propose that endocrine mechanisms play a central role in the effects of obesity on bone. Fat compartments have different effects, and SAT is the compartment most strongly associated with bone measurements. SAT was associated with low bone resorption, and regression models suggest that the lower resorption may be mediated by leptin. Finally, understanding the mechanisms associated with the effects of obesity on bone strength might help to identify new therapeutic strategies for osteoporosis.

## Declaration of interest

T V received grant and personal fees from Pharmacosmos. J S W has received speaker honoraria from Eli Lilly and Sandoz; grant funding from Alexion; donation of drug from Eli Lilly and Consilient for clinical studies; and consulting fees from Mereo Biopharma. R E receives consultancy funding from IDS, Sandoz, Nittobo, Samsung, Haoma Medica, CL Bio, Biocon, Amgen, Hindustan Unilever, Pharmacosmos, Takeda and Viking and grant funding from Nittobo, Roche, Pharmacosmos and Alexion. The other authors have nothing to disclose.

## Funding

This work was funded by a Biomedical Research Unit grant from the National Institute for Health Research http://dx.doi.org/10.13039/100005622. A E was funded by a PhD Studentship from the National Osteoporosis Society http://dx.doi.org/10.13039/501100000300 and Orthopaedic Research UK http://dx.doi.org/10.13039/501100000776.
